# Supplementary material for: Pharmacological Rescue of the Brain Cortex Phenotype of Tbx1 Mouse Mutants: Significance for 22q11.2 Deletion Syndrome
Source: Front Mol Neurosci. 2021 Sep 6;14:663598. doi: 10.3389/fnmol.2021.663598 (PMC8450345; doi:10.3389/fnmol.2021.663598)
Supplement: Supplementary file 1 [file Presentation_1.pdf]

*Cell counts on adult brain sections immunostained for TBR1:* Cryosectioning of adult brains and immunostaining for TBR1 were performed as described for embryonic brains. Cell counts were performed on coronal sections of a single hemisphere per animal (8 weeks of age, n = 5 / genotype / treatment) using counting boxes (500 µm wide) that were placed in the primary somatosensory cortex (S1) around Bregma level 0.50 mm (Paxinos and Franklin, 2012)*. Boxes spanned the entire thickness of the cortex (white matter to pia) and were divided into 12 equidistant bins. The average density of TBR1+ cells / 100 $\mu m^2$ for each bin was statistically analyzed and graphically represented using Microsoft Office Excel software. Results are expressed as the mean ± standard deviation (SD). The unpaired, 2-tailed Student's *t*-test and the $\chi 2$ test were used for statistical analysis. As the laminar distribution of labelled cells was measured, we applied the Benjamini–Hochberg procedure to correct for multiple testing. Values of $P \leq 0.05$ were considered to be statistically significant.

* Paxinos G, Franklin K. 2012. Mouse brain in stereotaxic coordinates. NewYork: Academic Press.
