# Supplementary figures and images for: Pharmacological Rescue of the Brain Cortex Phenotype of Tbx1 Mouse Mutants: Significance for 22q11.2 Deletion Syndrome
Source: Front Mol Neurosci. 2021 Sep 6;14:663598. doi: 10.3389/fnmol.2021.663598 (PMC8450345; doi:10.3389/fnmol.2021.663598)

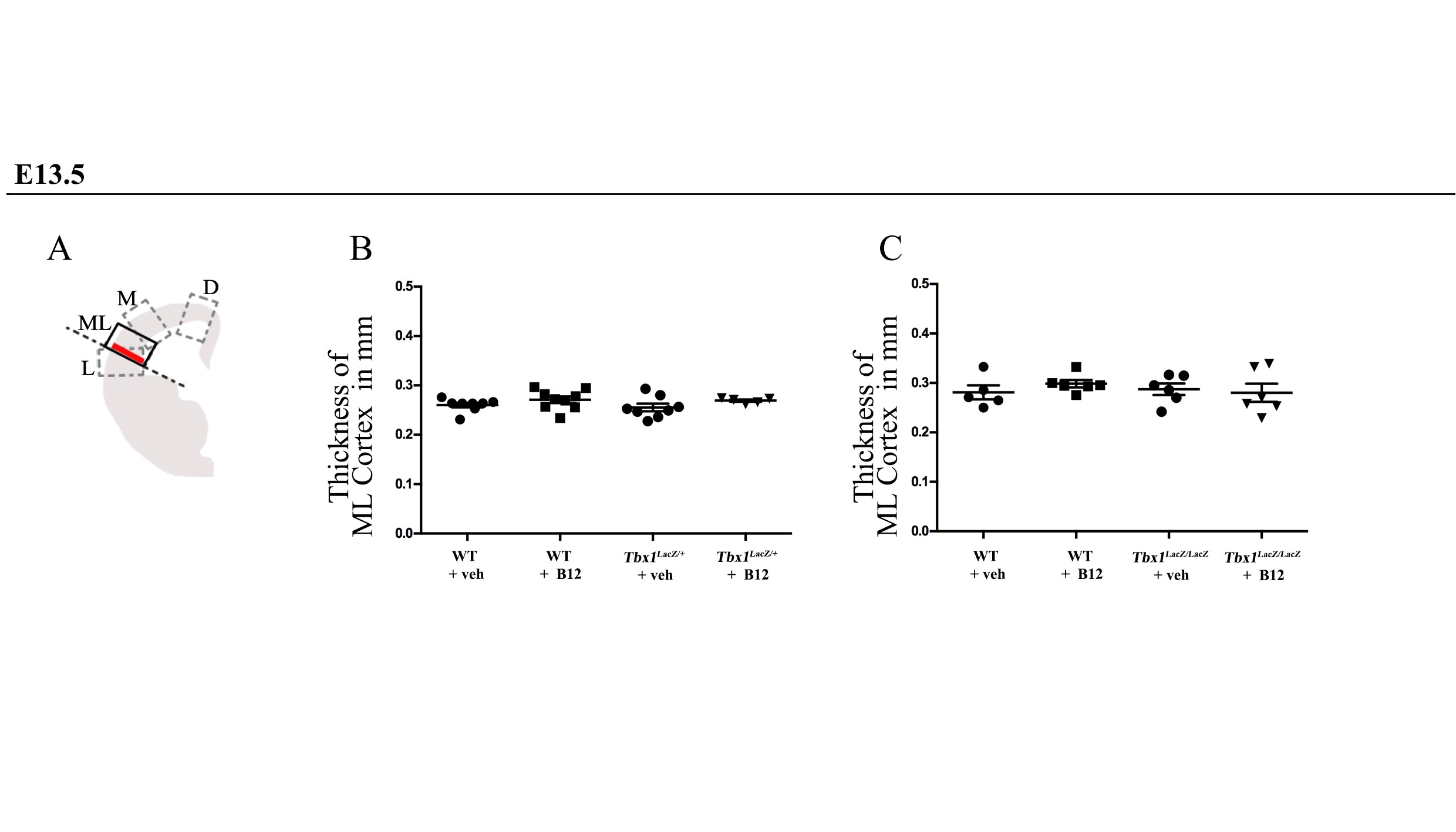

Supplement: Supplementary Figure 1 — Thickness of ML-cortex is not altered in Tbx1 mutant embryos at E13.5. The cartoon (A) indicates the position of the ML-cortex in coronal brain sections (boxed area) at E13.5. The red line indicates the position where cortical thickness (ventricular-to-pial surface) was measured. Cortical thickness was similar in all genotypes and treatment groups that included B12-treated and vehicle-treated Tbx1lacZ/+ embryos (B) or Tbx1lacZ/LacZ embryos (C). [file Image_1.JPEG]
